# Estimation of the Plastic Zone in Fatigue via Micro-Indentation

**DOI:** 10.3390/ma14195885

**Published:** 2021-10-08

**Authors:** Cristina Lopez-Crespo, Alejandro S. Cruces, Stanislav Seitl, Belen Moreno, Pablo Lopez-Crespo

**Affiliations:** 1 Department of Civil and Materials Engineering, University of Malaga, C/Dr Ortiz Ramos s/n, 29071 Malaga, Spain; crilop60@uma.es (C.L.-C.); ascruces@uma.es (A.S.C.); bmoreno@uma.es (B.M.); 2 Department, I. E. S. Politecnico Jesus Marin, C/Politecnico, 1, 29007 Malaga, Spain; 3 Institute of Physics of Materials, Czech Academy of Science, Žižkova 22, 61662 Brno, Czech Republic; seitl@ipm.cz; 4 Faculty of Civil Engineering, Brno University of Technology, Veveří 331/95, 60200 Brno, Czech Republic

**Keywords:** plastic zone in fatigue cracks, fatigue of materials, micro-indentation

## Abstract

Accurate knowledge of the plastic zone of fatigue cracks is a very direct and effective way to quantify the damage of components subjected to cyclic loads. In this work, we propose an ultra-fine experimental characterisation of the plastic zone based on Vickers micro-indentations. The methodology is applied to different compact tension (CT) specimens made of aluminium alloy 2024-T351 subjected to increasing stress intensity factors. The experimental work and sensitivity analysis showed that polishing the surface to #3 μm surface finish and applying a 25 g-force load for 15 s produced the best results in terms of resolution and quality of the data. The methodology allowed the size and shape of both the cyclic and the monotonic plastic zones to be visualised through 2D contour maps. Comparison with Westergaard’s analytical model indicates that the methodology, in general, overestimates the plastic zone. Comparison with S355 low carbon steel suggests that the methodology works best for alloys exhibiting a high strain hardening ratio.

## 1. Introduction

The plastic zone refers to the distance over which the material is plastically deformed at the tip of a growing crack subject to cyclic load. The size and shape of such a zone is a direct measure of the damage taking place at the tip of the crack. Accordingly, accurate knowledge of the evolution of the plastic zone might be extremely useful to quantify the damage in materials. For example, the size of the plastic zone might be used as a way to estimate the fracture toughness of materials [1]. In addition, the size of the plastic zone is also a powerful tool to understand the unexpected failure of components. For instance, it might throw some light over the magnitude of stresses before fracture based on the plastic zone. Moreover, the plastic zone might be used to understand micromechanisms that control the deformation at the tip and ultimately the fracture process and has been found to be proportional to the work required in generating new crack surfaces [2]. In addition, the plastic zone can be used to predict fracture instability, e.g., through the critical elastic energy release rate concept [3]. To obtain the K-R curve for a material (resistance to crack extension), the plastic zone size is often added to the physical crack length in order to compute the effective crack length [4]. Given the key role of the plastic zone for understanding the damage ahead of a fatigue crack, it seems logical to use the size and shape of the plastic zone as a gold standard to validate different types of numerical models based on 2D and 3D finite element methods [5,6,7], extended finite element methods [8,9] or crystal plasticity finite element modelling [10,11]. Such validation would clearly be more direct and hence more robust than using other fatigue and fracture parameters that can only be calculated after post-processing stress, strain or displacement data.

There exists a number of fatigue approaches aimed at predicting the failure under Small Scale Yielding (SSY) or Large Scale Yielding (LSY) conditions. For example, the Paris [12], Walker [13] or Forman [14] equations are in general more suitable for SSY conditions while the J-integral [15], J-Q theory [16], the J-A2 three-term approach [17] or the modified J-Q solution [18] were devised for LSY or fully plastic conditions. Accurate experimental characterisation of the plastic zone allows the transition between SSY and LSY to be identified and hence helps understanding why one or another method should be used under certain conditions. 

One method to characterise experimentally the plastic zone is to measure the hardness at different locations around the crack tip [19]. The volume of material within the plastic zone has been plastically deformed and thus has hardened. Such increment in hardness can be detected with indentations [20]. Such a method has been used in the past because it is straightforward and is based on direct measurements of a material property. Early works measured the hardness along a straight line, typically coinciding with the crack growing direction [21,22]. Such works employed around 20 measurement points to characterise the crack tip. Subsequent works focused on a number of issues both related to the material and the techniques, such as the effect of using a very low load, the effect of the microstructure [19], the effect of high temperatures on the plastic zone [23] or the influence of the specimen geometry on the plastic zone [24]. Nevertheless, their plastic zone characterisation was based on measuring the hardness along single directions. Subsequent works extended the methodology to four different directions [25,26]. Their measurements consisted of measuring the hardness radially from the crack tip. This allowed relatively large plastic zones to be studied but the data did not allow 2D maps to be generated. To the authors’ knowledge, a bi-dimensional study of the plastic zone has only been performed by Bhattacharyya and co-authors [27]. They generated 2D maps of the plastic zone on an M-50 NiL bearing steel. Unlike in the current work, such a plastic zone was not generated by a standard crack tip field and instead was referred to as the rolling contact fatigue affected plastic zone. 

All of the above-mentioned works are based on few hardness measurements and do not allow to properly characterise the plastic zone. In addition, none of the above works give enough experimental details so that the method can be reproduced in other materials and conditions. In this work, an improvement over the previous works is proposed. We perform an ultra-fine experimental characterisation of the plastic zone based on micro-indentations and propose a criterion for identifying the boundary between plastic and elastic behaviour. Moreover, plenty of experimental detail is given here so that the method can be reproduced in other laboratories provided they have any type of hardness method. First, the material is described, both in terms of main mechanical properties and microstructure. Then the experiments and the micro-indentation procedure are explained. This explanation is enhanced with a sensitivity analysis of the key experimental parameters that have an effect on the micro-indentation measurements for estimating the plastic zone. Finally, the micro-indentation results are mapped for the different specimens and these are compared with analytical models and discussed. 

## 2. Materials and Methods

### 2.1. Aluminium Alloy 2024-T351

**Figure 1:**
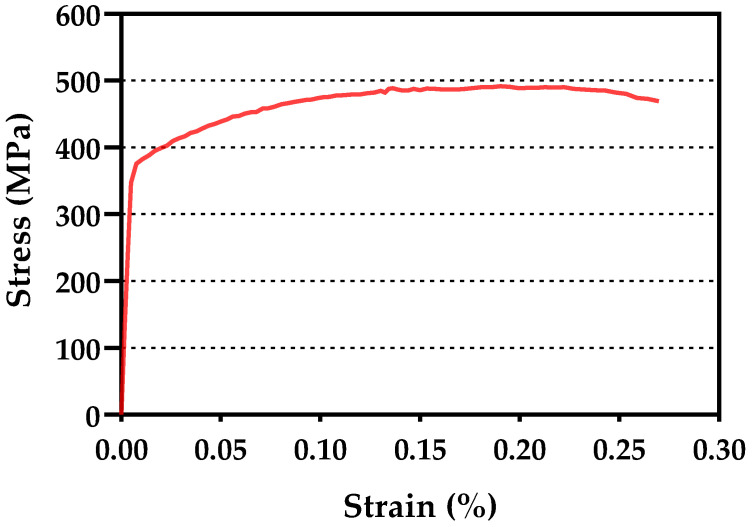
Monotonic stress–strain curve obtained with a tensile test.

**Figure 2:**
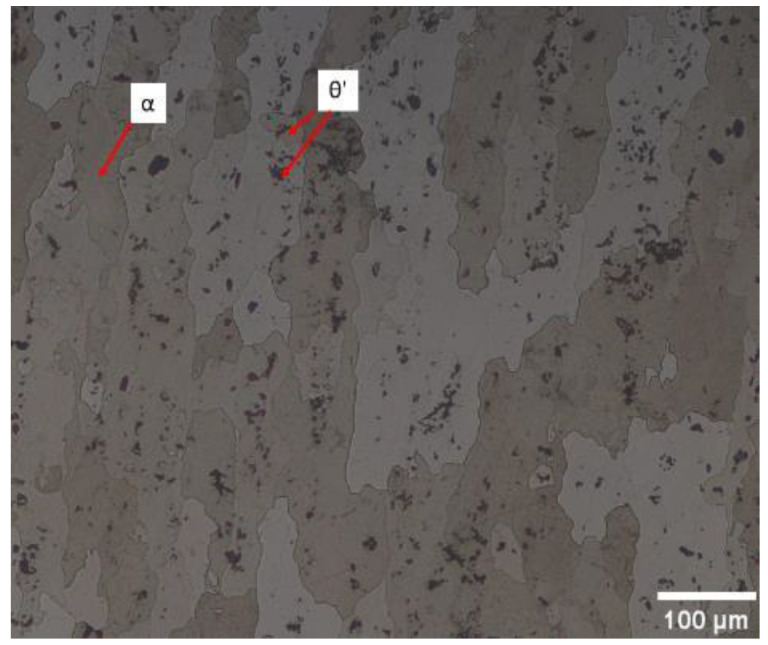
An optical micrograph of the 2024-T351 aluminium alloy showing the two principal phases.

2024-T351 aluminium alloy was used in this work. The chemical composition given by the manufacturer (Suministros Industriales Miguel Lopez S.L., Malaga, Spain) is summarised in Table 1. The tensile test curve of the material is shown in Figure 1. The mechanical properties of the alloy are given in Table 2 and the strain hardening ratio (σ_u_/σ_y_) is 1.31. A micrograph showing the microstructure is presented in Figure 2, with a grain size of approximately 74 μm being recorded [28]. The microstructure is mostly formed of α phase with θ’ intermetallic compound (AlCu_2_) as precipitates. These are consequences of the heat treatment applied to the alloy and cause the increase in strength and hardness in the alloy. The average size of the precipitates is 6.8 μm and 8.6 μm in the crack growing direction and in the crack opening direction, respectively.

### 2.2. Fatigue Experiment

**Figure 3:**
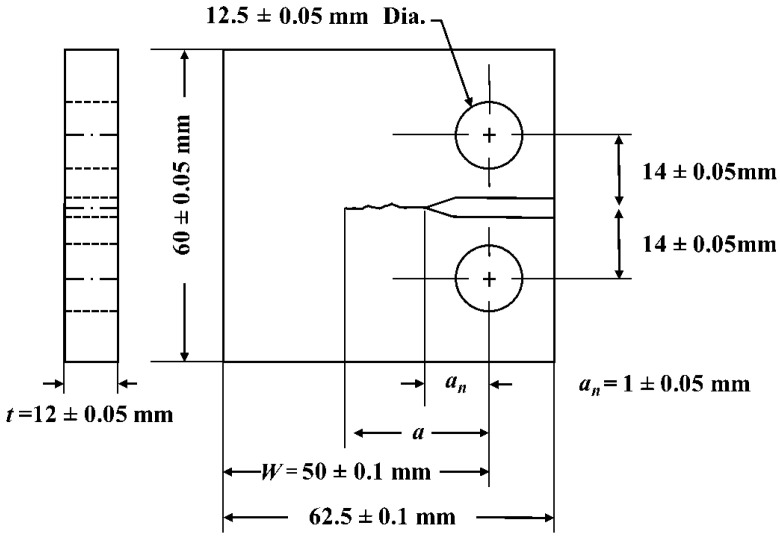
Compact tension (CT) geometry of the specimen, manufactured following ASTM standard [29].

Three Compact Tension (CT) specimens were used for the fatigue tests. A schematic of the specimen shape and dimensions is shown in Figure 3. The initial crack length (a_n_ in Figure 3) was 11 mm. The fatigue parameters are summarised in Table 3. The cyclic loading of the specimens was conducted on an Instron 8501 loading rig. The load ratio, R, was 0.1 for all specimens and the load frequency was 20 Hz throughout the tests. The maximum applied load was 2.1 kN in all cases. The different stress intensity factors (ΔK) were achieved by testing different crack lengths. The crack length was monitored with the help of a 5 MP CCD Limes camera. The specimens were subjected to constant load amplitude and increasing ΔK. The number of cycles required for achieving the crack length described in Table 3 were 40,300 cycles, 612,000 cycles and 646,000 cycles for specimens P1, P2 and P3, respectively. 

## 3. Micro-Indentation Technique 

### 3.1. Description of the Micro-Indentation Experimental Procedure 

The micro-indentation tests were conducted following the standards [30]. To this end, a Matsuzawa MXT-70 micro-hardness tester (Kawabe, Japan) was used. Vickers micro-hardness, HV, was measured with the tester, according to the following expression [30]:

$$HV=1854.4\frac{P}{d^{2}}$$

where *P* is the force applied with the micro-indenter and *d* is the average of the diagonals measured in the indentations. The diagonals in the indentation were measured with a 24 MP EOS 2000D digital camera coupled to a Nikon Epiphot 280 optical microscope (Champaign, IL, USA). This is a relatively low spec system. By using higher-end microscopes and imaging systems, the quality of the readings can be improved. Nevertheless, by using relatively simple arrangements such as the current set-up, the plastic zone analysis is more accurate than previous studies [20,23,25,26]. The measurements were made with ImageJ open-source software (version 1.40 g) [31].

**Figure 4:**
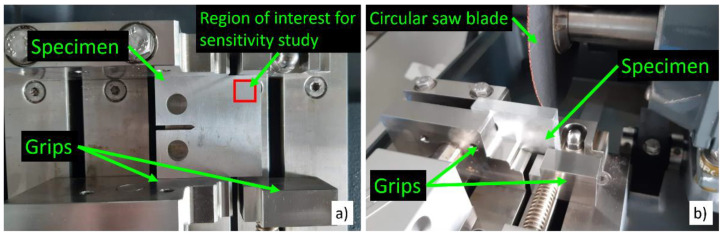
CT specimen ready to be cut with a circular saw blade. (**a**) The grip might induce a force in the crack opening direction. (**b**) The grip does not induce any load that might affect the fatigue crack plastic zone.

One of the advantages of the current procedure is its low cost yet allowing a high resolution to be obtained. In order to achieve the highest possible resolution, the region of the crack tip in the specimen needs to be polished to a submicron grade. It is very difficult to achieve such a fine finish on the entire surface of the CT specimen. Consequently, it is necessary to cut a fragment of the specimen. This is in contrast to recent works where the plastic zone was estimated with indentation measurements directly taken on the specimen [26]. Such a fragment should be noticeably larger than the plastic zone so that the procedure does not have an effect on the estimation. Special care must be taken when cutting the specimen so that no additional plasticity is introduced in the specimen. In the current arrangement, the fragment was obtained by cutting it with a circular saw blade. The set-up required the specimen to be firmly held and the grip of the specimen is obtained with a lever (see Figure 4). A very strong grip in the crack opening direction near the holes (see Figure 4a) might induce some plastic deformation around the crack tip. The solution used here was to place the specimen vertically (Figure 4b) so that the grip force does not induce any load along the crack opening direction.

According to the standard [30], the full force should be applied between 10 and 15 s. When applying the full test force for 10 s the overall hardness was lower (smaller indentations) than for 15 s. This in turn made the shape of the plastic zone somewhat blurred and was detrimental for estimating the size of the plastic zone. As a consequence, the load was applied for 15 s for all hardness measurements. 

### 3.2. Sensitivity Analysis 

**Figure 5:**
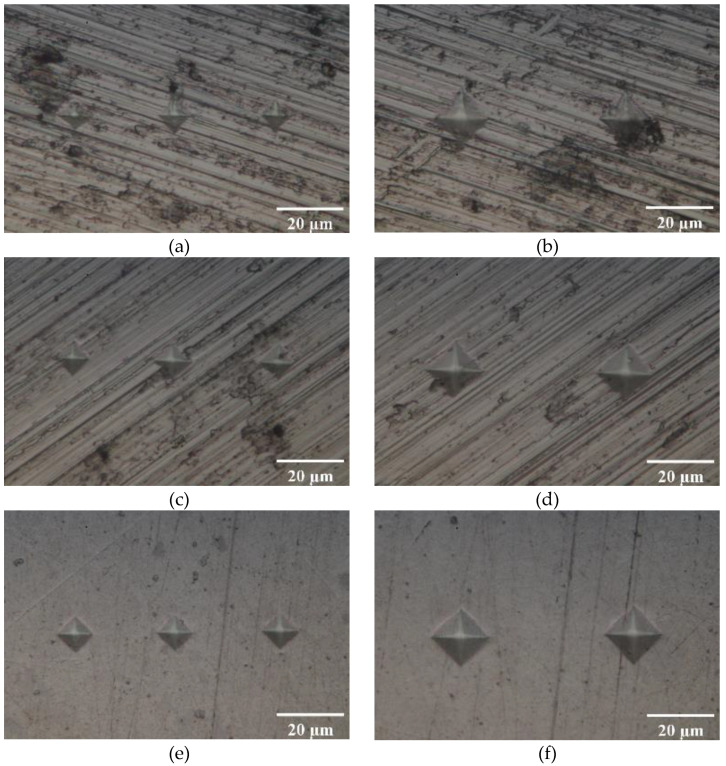
Different indentations as observed in different surface finish. (**a**) #800 surface finish and indentation obtained with 10 gf, (**b**) #800 and 25 gf, (**c**) #1200 and 10 gf, (**d**) #1200 and 25 gf, (**e**) #3 μm and 10 gf, (**f**) #3 μm and 25 gf. All images were taken on a Nikon Epiphot 280 optical microscope with a 100× lens.

In order to achieve good accuracy for the estimation of the plastic zone, the hardness measurements should be as close together as possible. In addition, the load that is applied with the hardness tester directly affects the resolution for the estimation of the plastic zone. On the one hand, a higher load produces indentations with a better signal to noise ratio in each HV measurement. That is, the higher the load, the better the quality of each HV. On the other hand, applying higher loads produces larger indentations. The distance between adjacent indentations should be larger than 2.5 times the length of the indentation diagonal to avoid interference between consecutive measurements [30]. Therefore, using a higher force implies a lower resolution in the estimation of the plastic zone since the number of HV measurements per unit surface is smaller, as compared to a lighter force. At the same time, lighter forces will produce smaller indentations and therefore the surface preparation becomes more and more critical [30]. Hence, a parametrical study was performed in order to identify a good compromise in terms of indentation force, surface finish and accuracy for identifying the plastic zone. To this end, series of measurements were taken along the crack opening direction and along the crack growing direction. Each series was taken in a straight line from a region removed from the plastic zone to avoid the influence of the crack tip plasticity. This is shown in Figure 4a as “Region of interest for sensitivity study”. The same distance in the material was covered with 10 indentations taken at 25 gf and with 15 indentations taken at 10 gf. Three different surface finishes were studied. These were #800, #1200 and #3 μm, with #3 μm being the finest surface finish among the different cases studied. The #800 and #1200 surface finishes were obtained with #800 grade and #1200 grade SiC abrasive papers, respectively. #3 μm finish was obtained by pre-charging the polishing cloth with 3 μm grade diamond in a carrier paste. The cloth was also moistened with emulsifying fluid during the polishing. In addition, two different loads were tested, namely 10 gf and 25 gf (1 gf is 9.8 × 10^−3^ N. Gram-force is the force unit normally used in micro-indentation [30]). Some examples showing the different surface finishes are shown in Figure 5. The #800 and #1200 surface finishes were used to understand the effect of poor surface preparation on the micro-indentation procedure. Figure 5 also shows different indentations as obtained with the lower and the higher force. The improvement of the surface finish is clear from the different images shown in Figure 5. The larger size of the indentations produced with 25 gf is also evident in the images shown in Figure 5. Such larger indentation implies a smaller number of measurements taken over the same distance, as seen in Figure 5. 

The #800 surface finish (Figure 5a,b) shows very irregular indentations, especially under 10 gf (Figure 5a), where the difference between indentations is greatest and the difference between vertical and horizontal diagonal is large. When the difference between the diagonals is greater than 5%, the indentation should be discarded [30]. In addition, the poor surface finish creates dark regions that make it difficult to measure the diagonals and, therefore, decreases the quality of the hardness measurements. In some cases, these dark regions obscure the indentation to the point that the measurement of the diagonal is not possible. Comparison between images taken at 10 gf (Figure 5a,c,e) and images taken at 25 gf (Figure 5b,d,f) clearly shows more deformed indentations with decreasing load. Moving from #800 to #1200 clearly improves the quality of the indentations, even though they are still quite irregular, particularly under 10 gf (Figure 5c). The distortion of the indentations is clearly reduced by polishing to the next grade (#3 μm), as observed in Figure 5e,f. Moreover, the polishing direction is drastically reduced by using 3 μm grade diamond in a carrier paste (see Figure 5e,f). The finest polish (Figure 5e,f) produces the most regular indentations and the most uniform background, free of features, thus making it easier to measure the indentations diagonals. 

**Figure 6:**
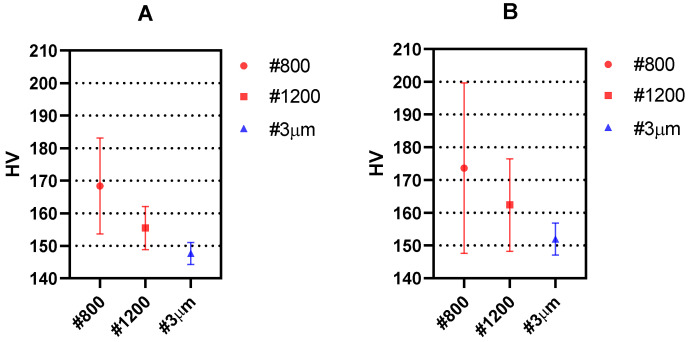
Mean hardness measurements obtained with Vickers micro-hardness testing along the crack growing direction for different surface finish and (**A**) 25 gf and (**B**) 10 gf. The standard deviation is shown as error bars.

**Figure 7:**
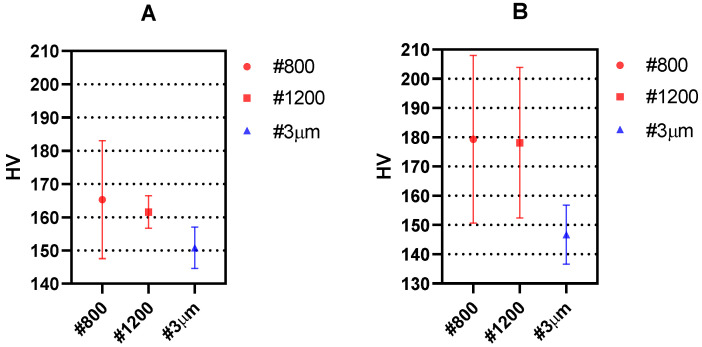
Mean hardness measurements obtained with Vickers micro-hardness testing along the crack opening direction for different surface finish and (**A**) 25 gf and (**B**) 10 gf. The standard deviation is shown as error bars.

The reproducibility of the measurements is studied by plotting the mean and the standard deviation for the different experimental conditions. The results of the measurements along the crack growing direction and along the crack opening direction in a region away from the crack tip are shown in Figure 6 and Figure 7, respectively. 

Figure 6 and Figure 7 show that both the mean values as well as the scattering (measured as standard deviation) in the measurements decrease as the surface finish is improved. This is due to the easier surface penetration of the indenter due to less surface irregularities existing in the surface. Such behaviour is in agreement with previous works where higher scattering was recorded for smaller loads of the indenter [19]. The fact that larger indenter loads produce overall lower HV values has also been observed in previous repeatability studies [30]. Such effect is observed here in the different surface finishes under study. Accordingly, the best results both in the crack growing direction (Figure 6) and in the crack opening direction (Figure 7) are obtained with 25 gf load and #3 μm surface finish. 

## 4. Results and Discussion

### 4.1. Plastic Zone Estimation 

**Figure 8:**
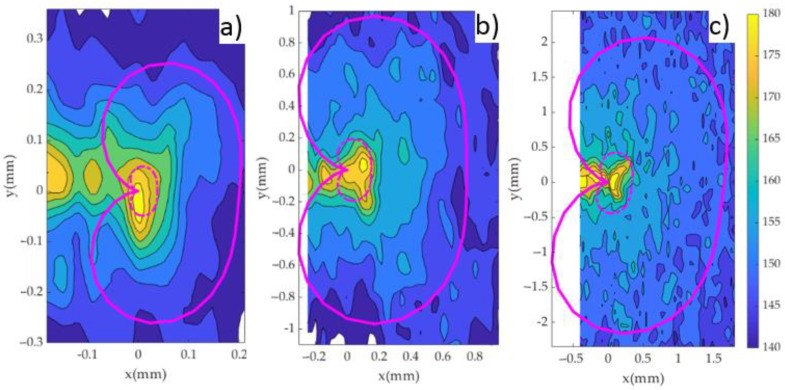
2D contour maps of micro-hardness around the crack tip for the three specimens: (**a**) P1, ΔK = 9.91 MPa√m, (**b**) P2, ΔK = 20.61 MPa√m and (**c**) P3, ΔK = 30.23 MPa√m. The crack tip is located at coordinates (0, 0) in all maps. The Westergaard theoretical prediction of the monotonic plastic zone is shown as a pink solid line. The theoretical cyclic plastic zone is shown as a pink dashed line.

Figure 8 shows the 2D contour maps of micro-hardness measurements taken around the crack tip for the three specimens. Following the results of the previous section, all indentations were performed with 25 gf load and #3 μm surface finish. The number of data points collected was 322, 1075 and 4365 for samples P1, P2 and P3, respectively. Such an amount of data points appears to be sufficient to properly characterise the Vickers hardness evolution around the crack tip. Hence, the resolution achieved is up to 57 times higher than in previous works [20,23,25,26]. The data were linearly interpolated to generate the contour plots. In addition, the data were smoothed with a Gaussian filter operator. This is a 2D convolution operator that provides gentler smoothing than other operators and preserves features better than other operators [32]. Figure 8a–c show the results for samples P1, P2 and P3, respectively. For validation purposes, the theoretical monotonic plastic zone was also computed in a similar fashion to previous works [33]. This theoretical estimation was based on the Westergaard stress field around the crack tip (Equations (1)–(4)) [34] combined with the Von Mises criterion [35]. Such a solution is well established to understand the shape and size of the plastic zone [33,35,36]. Plane stress conditions were assumed since the analysis is performed at the surface.

(1) $$\sigma_{xx}=A_{0}\cdot r^{-\frac{1}{2}}\cdot \cos\left(\frac{\theta }{2}\right)\cdot \left(1-\sin\left(\frac{\theta }{2}\right)\cdot \sin\left(\frac{3\theta }{2}\right)\right)$$

(2) $$\sigma_{yy}=A_{0}\cdot r^{-\frac{1}{2}}\cdot \cos\left(\frac{\theta }{2}\right)\cdot \left(1+\sin\left(\frac{\theta }{2}\right)\cdot \sin\left(\frac{3\theta }{2}\right)\right)$$

(3) $$\tau_{xy}=A_{0}\cdot r^{-\frac{1}{2}}\cdot \cos\left(\frac{\theta }{2}\right)\cdot \sin\left(\frac{\theta }{2}\right)\cdot \cos\left(\frac{3\theta }{2}\right)$$

(4) $$A_{0}=\frac{K_{I}}{\sqrt{2\cdot \pi }}$$

where *r* and *θ* are the polar coordinates with respect to the crack tip and K_I_ is the applied stress intensity factor. 

The cyclic plastic zone was also computed analytically and used as a reference for comparison. The theoretical cyclic plastic zone was also calculated, according to [37]:

(5) $$r_{c}\left(\theta \right)=\frac{1}{4\pi }{\left(\frac{\Delta K_{I}}{2\sigma_{y}}\right)}^{2}\left[1+\cos\theta +\frac{3}{2}\sin^{2}\theta \right]$$

where *r_c_* and *θ* are the polar coordinates values of the cyclic plastic zone boundary, ΔK_I_ is the stress intensity factor range in mode I and *σ_y_* is the yield stress.

These theoretical plastic zones are also plotted in Figure 8 as a pink solid line (monotonic) and pink dashed line (cyclic). The crack tip is located at the origin of coordinates (coordinates (0, 0) in Figure 8). The plastic zones were tilted slightly in Figure 8a,c to compensate for the crack angle observed experimentally in samples P1 and P3. For sample P2 the crack appeared perfectly horizontal at the time of analysis, so no angle correction was required in this case. As observed in Figure 8, the HV scale starts at 140 HV and ends at 180 HV, with 5 HV steps. Based on the sensitivity analysis (Figure 6 and Figure 7), hardness values at 145 HV or below could be considered as non-affected by the crack tip hardening [38]. 

The data gathered away from the plastic zone was useful to identify 145 HV as the threshold value above which the material hardens. Accordingly, the 145 HV contour can be used to set the limits of the monotonic plastic zone. Figure 8 shows an acceptable agreement, both in size and shape between the monotonic plastic zone estimated by micro-indentation and the theoretical monotonic plastic zone estimated according to Westergaard’s solution, with Westergaard’s estimation generally underestimating the size of the plastic zone, as compared to the experimental results. The shape of the micro-hardness contours roughly agree with the boundary indicated by Westergaard’s solution. It is also observed that hardness measurements are affected by some noise (Figure 8). This noise is probably caused by the naturally uneven distribution of θ’ precipitates (harder phase) as observed in Figure 2. The fact that the size of the indentations is similar to the size of the θ’ precipitates will most likely contribute to the distortion of the HV contours in Figure 8. The noise is probably the reason why Westergaard’s estimation of the plastic zone is closer to the 150 HV contour for P2 and P3, while for P1 appears to be closer to the 145 HV contour (Figure 8). Regarding the cyclic plastic zone, the theoretical estimation falls around the 170 and 175 HV contours. 

**Figure 9:**
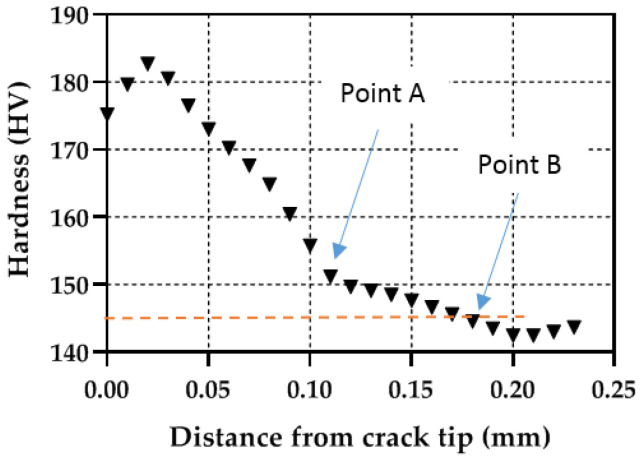
Micro-hardness profile taken along the crack growing direction for simple P1 (ΔK = 9.91 MPa√m).

**Figure 10:**
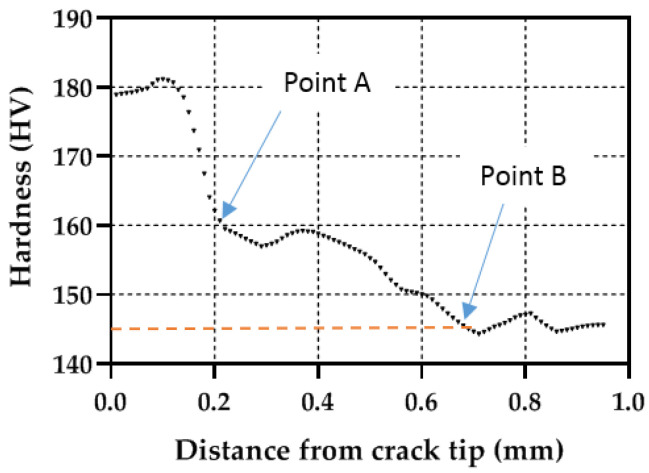
Micro-hardness profile taken along the crack growing direction for simple P2 (ΔK = 20.61 MPa√m).

**Figure 11:**
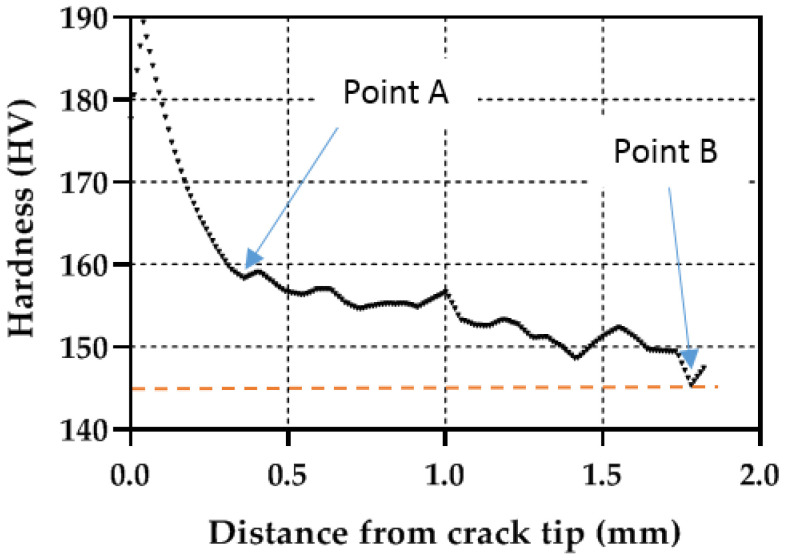
Micro-hardness profile taken along the crack growing direction for simple P3 (ΔK = 30.23 MPa√m).

An additional analysis to compare the plastic zones can be performed by extracting the hardness profiles along the crack growing direction (X direction in Figure 8). Figure 9, Figure 10 and Figure 11 show the profile results for samples P1, P2 and P3, respectively. All HV profiles show an initial short increase in HV just ahead of the crack tip. Beyond this maximum, the data show two parts: an initial portion with a high negative slope followed by a lower negative slope region. For sample P2 (Figure 10), the initial maximum is located at X = 0.10 mm. The first high slope takes place between X = 0.10 and X = 0.21 mm. Finally, the third region with a lower negative slope takes place at X > 0.21 mm. A similar trend is clearly observed in the three specimens. The similar behaviour in the three specimens cross-validates the current methodology. The two different slopes were also identified in different materials [20,23,25,26]. The initial surge just ahead of the crack tip was also detected by Ghodrat and co-authors on a 6061-T4 aluminium alloy [20]. The boundary between the larger and the smaller slope was used as the locus for the cyclic plastic zone, following earlier works by Gwider and Ranganathan [26]. This is identified in Figure 9, Figure 10 and Figure 11 as “Point A”. Gwider and Ranganathan also defined the boundary of the monotonic plastic zone as the end of the second lower slope. However, such a criterion for defining the monotonic plastic zone boundary appears somewhat imprecise for our data. Instead, we propose using the first time that the data hits 145 HV as the boundary for the monotonic plastic zone. Such demarcation seems more unequivocal and hence easier to replicate for other alloys and conditions. 145 HV is chosen because such value was previously identified as the threshold value above which the material hardens. This boundary for the monotonic plastic zone is highlighted in Figure 9, Figure 10 and Figure 11 as “Point B”. The 145 HV threshold is also shown as a red dashed line in Figure 9, Figure 10 and Figure 11. The scattering of the data appears to be lower for sample P1 (Figure 8), followed by sample P2 (Figure 9), with the most scattered data being those of sample P3 (Figure 10). Such difference could be linked to different size regions of interest being analysed for samples P1, P2 and P3 (see differences in X-axes in Figure 8, Figure 9 and Figure 10). The region of interest was largest for sample P3 as a larger plastic zone was expected. This, in turn, makes more hard phases to be within the region of interest making the profile fluctuating more. 

The plastic zone size results together with the error (in %) and the differences (in mm) with respect to the theoretical and the numerical plastic zone are summarised in Table 4. It is observed that the experimental estimation of the monotonic plastic zone is good for the three samples, with the largest error being 10% for sample P1. Table 4 clearly shows that the experimental estimations are better for the monotonic plastic zone than for the cyclic. In addition, the error in the estimation of the cyclic plastic zone decreases as the plastic zone increases. This suggests that the accuracy of the methodology for the alloy under study might be around 0.2 mm in the sense that plastic zones (both monotonic and cyclic) of 0.2 mm or larger seem to be well characterised. Following the previous discussion, this accuracy is influenced by the relative size of the indentations with respect to microstructural features and the size of the plastic zone. By looking at the difference between the theoretical and the experimental estimations (last column in Table 4) it is clear that the dissimilarity is not as large as it might initially appear by noting the cyclic error for samples P1 and P2. Previous analyses suggest that there should be between 10 and 100 characteristic microstructural features within the plastic zone so that the material can be considered a homogeneous continuum [39,40]. Taking into account the microstructure described in Section 2.1, it appears that at least 25 different orientations (or characteristic microstructural features) is the minimum number required for the method to give sensible results. For smaller plastic zones, or for materials with larger microstructural features, the methodology seems to not give sensible results in terms of plastic zones. Accordingly, the accuracy of the methodology herein described is clearly dependent on the microstructure.

### 4.2. Preliminary Analysis on S355 Steel

**Figure 12:**
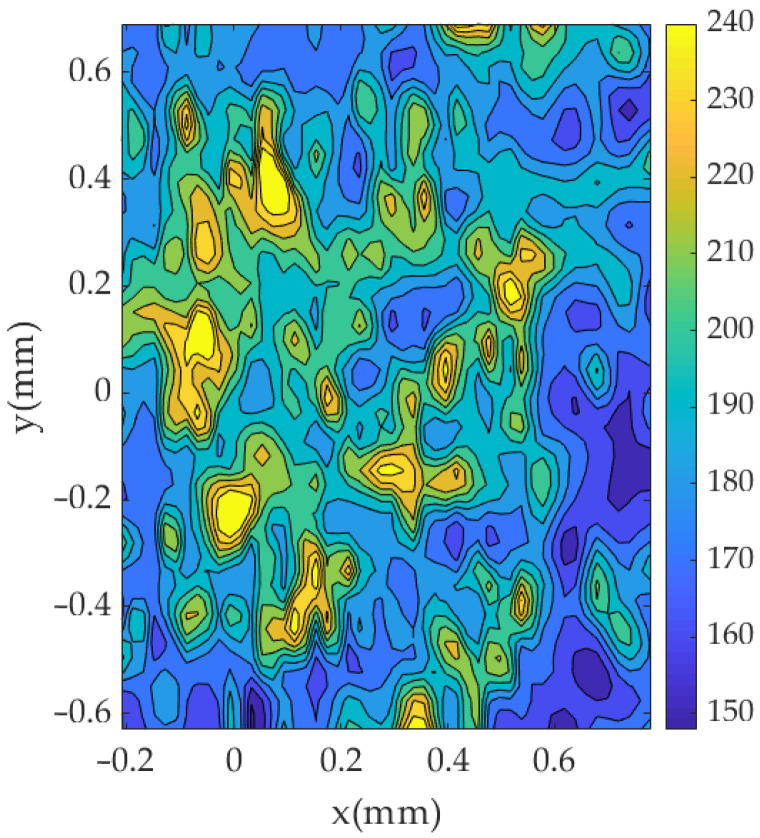
2D contour map of micro-hardness around the crack tip in a S355 steel. The crack tip coordinates are located at (0, 0).

A preliminary analysis was also performed on a S355 low carbon steel. The same procedure as that described in Section 3 is followed. Figure 12 illustrates a typical 2D contour map of micro-hardness distribution around the crack tip. The contour map is clearly noisier than that observed for the 2024-T351 aluminium alloy. The hardness variation across the maps makes it very difficult to identify the plastic zone. The S355 steel employed for this analysis has a strain hardening ratio of 1.11. This is considered a low hardening ratio for metals [41]. The results were consistently more inaccurate than on the 2024-T351 aluminium alloy. This is probably due to the hardness measurement being highly dependent on the hardening behaviour of the material [30]. A lower hardening ratio produces lower quality prints for retrieving the hardness and thus the overall visualisation of the plastic zone becomes more difficult. The higher hardening ratio of the aluminium alloy 2024-T351 (1.31) improves the signal to noise ratio and yields a better map for estimating the plastic zone, as observed by comparing Figure 8 and Figure 12. Based on the results from these two materials, it seems that the methodology provides reliable results for materials exhibiting strain hardening ratios of 1.3 or higher. Higher strain hardening ratios would give rise to larger hardness differences between the material within the plastic zone and the material away from the plastic zone and this will produce clearer plastic zone boundaries.

## 5. Conclusions and Future Research

A methodology based on Vickers micro-indentation for characterising the monotonic and the cyclic plastic zone has been described. It is based on the analysis of 2D contour maps of the hardness evolution around the crack tip. The methodology has been successfully applied on an aluminium alloy 2024-T351 subjected to three different ΔKs, namely 9.91, 20.61 and 30.23 MPa√m. The alloy exhibits a relatively high strain hardening ratio. The contour maps have allowed the plastic zone to be visualised. The experimental procedure has been described in great detail so that the technique can be replicated easily for other alloys or conditions. The main findings can be summarised as follows:

- The effect of the microstructure has been identified as the main cause of noise for the results. The fact that the size of the indentations is very similar to the hard precipitates of this alloy most likely decreases the quality of the results.
- The results and the sensitivity analysis indicate that for the current alloy, the best results are obtained by achieving a #3 μm surface finish, using 25 gf load for 15 s. Those parameters provide a good trade-off in terms of resolution and quality of each micro-indentation to produce a good enough map that can be used for quantitative analysis of plastic zones.
- The results have also shown that the shape and size can be approximately estimated from the 2D contour maps, as compared to Westergaard’s estimation of the plastic zone. The hardness profiles along the crack growing direction have allowed acceptable estimations of the plastic zone to be made, both for the monotonic and the cyclic plastic zones. For the cases studied, the estimations of the monotonic plastic zones have overall been better than those of the cyclic plastic zone.
- The results indicate that 0.2 mm appears to be the approximate size of the plastic zone that this methodology captures reliably. Plastic zones well below this figure can be spotted but visualisation of size and shape can be difficult.
- Based on previous works, a new criterion is proposed to separate the elastic from the plastic contribution from the micro-hardness measurements which enables us to retrieve the monotonic and the cyclic plastic zone. The current procedure for inferring the plastic zone around a fatigue crack tip is based on the hardening of the material being tested. Accordingly, applying this procedure to materials experiencing low strain hardening will yield less accurate results.

Part of the novelty of the current methodology lies in the combination of experimental data taken at the micro-scale for generating global parameters (plastic zone size). We believe this work contributes to bridging the gap between microstructural and continuum mechanics approaches for design against fatigue.

The results described in this work can be used to incorporate microstructural features into numerical simulations via micromechanical modelling [10,11,42]. Such a type of analysis can enhance the microstructural side of the current study regarding the plastic zone. 

## Figures and Tables

**Table 1:** Chemical composition in weight % of 2024-T351 aluminium alloy, provided by the manufacturer (the balance is Al.).

Mn	Si	Cr	Cu	Zn	Pb	Fe	Ti	Mg
0.650	0.070	0.010	4.570	0.060	0.004	0.120	0.039	1.500

**Table 2:** Monotonic properties of 2024-T351 aluminium alloy as obtained from the tensile test.

**Yield Stress, σ_y_**	375 MPa
**Ultimate Tensile Stress, σ_u_**	492 MPa
**Young’s Modulus, E**	73 GPa

**Table 3:** Fatigue parameters used in the three specimens.

Specimen ID	a, mm	ΔK_I_, MPa√m
P1	30.40	9.91
P2	37.61	20.61
P3	40.25	30.23

**Table 4:** Summary of plastic zone sizes estimated experimentally with micro-indentation (PZ_exp_) and theoretically computed following Westergaard’s model (PZ_theo_). The error and difference of the estimations with respect to the theoretical results are also shown.

Sample	Type of PZ	PZ_exp_ (mm)	PZ_theo_ (mm)	Error (%)	Difference (mm)
P1	Cyclic	0.11	0.04	175	0.07
	Mono	0.18	0.20	−10.0	−0.02
P2	Cyclic	0.21	0.15	40.0	0.06
	Mono	0.69	0.75	−8.00	−0.06
P3	Cyclic	0.35	0.34	2.94	0.01
	Mono	1.78	1.64	8.54	0.14

## Data Availability

Authors are happy to share the data with anyone interested in further post-processing the data.
